# Sexual Orientation in Twins: Evidence That Human Sexual Identity May Be Determined Five Days Following Fertilization

**DOI:** 10.7759/cureus.51346

**Published:** 2023-12-30

**Authors:** John Hayman, Denys W Fortune

**Affiliations:** 1 Clinical Pathology, The University of Melbourne, Melbourne, AUS; 2 Gynecologic Pathology, Retired, Melbourne, AUS

**Keywords:** mtdna haplogroups, handedness, hamilton's rule, kin selection, gender dysphoria, mitochondria, sexual orientation, monozygous twins, sexual orientation twins, homosexuality

## Abstract

Human same-sex sexual attraction has been recorded from the beginning of written history. It remains a controversial topic, but recent theories favor prenatal influences. A paradox is the occurrence of same-sex orientation in twins in that there is a higher level of concordance in monozygous twins compared to that in dizygous twins or non-twin siblings. If sexual orientation was entirely genetically determined monozygous twins would be expected to have identical sexual inclinations. Monozygous twins have twice the incidence of sexual concordance in comparison to dizygous twins but a third of these pairs have different sexual identities. An explanation for this disparity may lie in the time an embryo splits to form two separate fetuses. If splitting occurs early in twin development each twin may develop his or her own sexual identity; splitting occurring later results in twins that have the same sexual dispositions.

A possible process for such determination may be in the mitochondria, with universal maternal inheritance of a proportion of normal functioning but alternate mitochondria. Variation in the distribution of these mitochondria in neural precursor cells becomes a mechanism for the development of intrinsic sexual orientation and for the spectrum of human sexual inclinations.

The timing of embryonic splitting may be determined from the examination of fetal membranes, and the concept of early fetal sexual orientation is open to support or disproval.

## Introduction and background

Human same-sex sexual orientation

Human same-sex sexual orientation has occurred in all races and at all times since first recorded history [1]. There is reason to believe that it has been present since our evolution as a species, even present in our hominid forebears [2]. The factors resulting in same-sex sexual orientation remain controversial but there is growing evidence indicating that sexual orientation is heavily influenced by prenatal biological mechanisms rather than by unidentified factors in post-natal socialization [3]. 

Post-natal socialization theories, however, persist. They include various proposals that homosexuality results from problems in relationships with parents, especially the parent of the same sex, during childhood. The proposals arise generally from a psychoanalytic tradition, based upon theories that are often not amenable to scientific testing. The following quote is from a representative paper: "Notably, the combination of overconcernedness of the mother and detachment and hypercriticism of the father push the boy into avoidance of 'masculine' behavior, which in turn leading to a feeling of inferiority because the boy considers himself as lacking in manliness" [4].

Other theories that have been proposed include maternal stress during pregnancy and diminished testosterone in pregnancy and the neonatal period due to hyperprolactinemia [1,5]. These, along with others, appear to have faded and no longer appear in subject reviews. The concept favored here, despite dissent, is that homosexuality was and perhaps still is of kin survival value and is universally inherited as a chance variation [6].

Twin pregnancy

**Figure 1:**
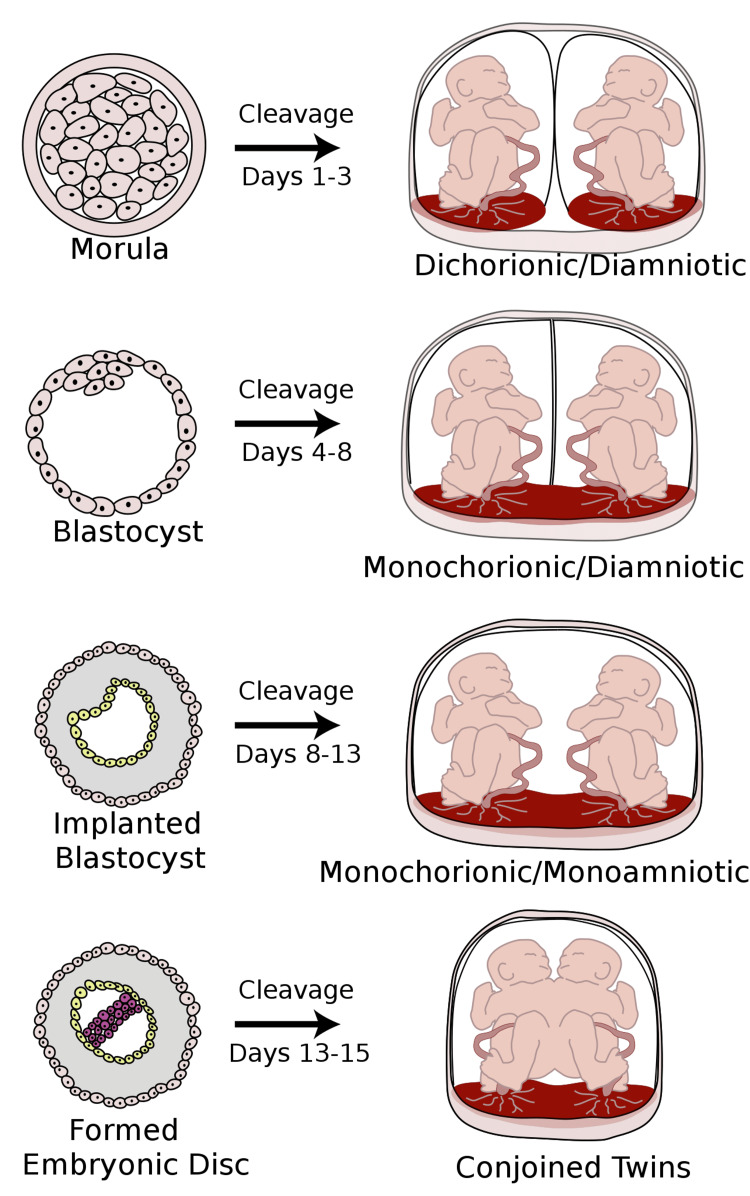
Formation of dichorionic/diamniotic, dichorionic/monoamniotic, and monochorionic/monoamniotic embryos depending on the time of zygote splitting 25-30% of splitting occurs within four days, 70% to 75% between four and eight days, and only 1% to 2% after day eight [6]. Dufendach K, (Artist). (2008). Placentation. Retrieved from http://commons.wikimedia.org/wiki/File:Placentation.svg

Twining may occur when two separate ova are fertilized at the same time, resulting in dizygotic (DZ) or fraternal twins [7]. These twins may be of different genders; if both are of the same gender the incidence of concordant sexual identity is the same as for non-twin siblings [8,9]. Alternatively, twinning may be monozygotic (MZ), which occurs when a single fertilized egg develops to the morula or blastocyst state but then subsequently splits into two embryos. Such cleavage may occur at any time from day one after fertilization up until thirteen days (Figure 1). Cleavage after thirteen days tends to be partial, resulting in the rare occurrence of conjoined twins.

The time of cleavage may be estimated from examination of the fetal membranes and placenta (chorionicity) [7]. Cleavage between days one and three results in twins each with their own chorion and amnion, dichorionic/diamniotic (DCDA) MZ twins, usually with separate placentas. Cleavage between days four to eight results in twins each with their own individual amnions but sharing the same chorion; monochorionic/DA (MCDA) MZ twins. The relatively rare cleavage after eight days but before 13 days results in MC/monoamniotic (MCMA) twins (Figure 1). If occurring after 13-14 days, conjoined twins may result.

Mitochondria

Mitochondria are present in all nucleated animal cells. They produce most of the energy requirements for a cell, energy in the form of ATP. Their number varies between several hundred to tens of thousands per cell, depending on that cell’s energy requirements [10]. Mitochondria are unique among animal cell organelles in that they contain their own DNA, mtDNA. MtDNA, like nuclear DNA (nDNA), is subject to mutation, mutation occurring more frequently than with nDNA [11].

Mutations that do not appreciably diminish mitochondrial function are described as non-pathological, adaptive, or functional variants; pathological mutations are changes to the mtDNA that reduce mitochondrial function. Adaptive variants occurring over the millennia have resulted in numerous mtDNA haplogroups, variations in the DNA that reflect racial origins, and descension from a common maternal ancestor [12]. Significant biochemical differences have been reported in mtDNA haplotypes [13].

As a result of mutations, cells may contain a mixture of mitochondria with normally functioning DNA (wild type) together with mutant DNA, referred to as heteroplasmy. When a cell divides mitochondria flow in equal numbers into daughter cells, but the proportion of mitochondria with the mutant DNA is random. Mutant, including adaptive mutant levels, in daughter cells may be different. As a result, the daughter cells vary in their levels of heteroplasmy, and this difference may be amplified with subsequent cell divisions [10].

As well as ATP production, mitochondrial functions influence various cellular metabolic pathways and hormone production [10]. They also are an intrinsic part of the mechanism that initiates individual cell death (apoptosis) and the timing of cell division. Mitochondrial function deficiency results not only in identified mitochondrial disorders but has been implicated in the pathogenesis of neurological degenerative diseases and cognitive decline [14]. Mitochondria may also have a role in the occurrence of psychiatric disorders, including depression, bipolar disorder, and schizophrenia [15].

## Review

Frequency of homosexuality

The frequency of same-sex orientation appears similar throughout different human population groups [16]. The existence of apparent racial or geographical differences and apparent differences over time may relate to social acceptance and clustering rather than true variation. No reports of variation in the frequency of same-sex orientation have been found within the different HLA or mtDNA haplogroups.

In general, men seem to be more category-specific in their sexual orientation while women on the other hand are more likely to demonstrate sexual fluidity [3]. The estimates of exclusive or preferred same-sex sexual inclination have varied between different studies. At one extreme, the early Kinsey reports gave an incidence of 10% (males exclusively homosexual for three years or more) [17,18]. A more representative survey has estimated an overall prevalence of predominant non-heterosexual sexual orientation as 2.8% of a USA population group [7]. A particularly valuable conclusion of the Kinsey group was that in human populations there is continuous variation in the degree of expression of same-sex preference, with categorization given on a seven-point scale from zero to six. On this scale zero is exclusively heterosexual; six is exclusively homosexual. The conclusion that sexuality does not fit into two strict categories and the contributions of the Kinsey group have been acknowledged [19].

The probability of a boy growing up to be homosexual increases for each older brother born to the same mother, the so-called fraternal birth order (FBO) effect. The initial investigation indicated that each older brother increased the probability of being homosexual by about 33% [20]. Despite repudiation, this finding has been confirmed with subsequent detailed analysis [21]. Although less than the FBO effect, there is an increased probability that, regardless of birth order, a same-gender sibling of a non-heterosexually orientated family member will have a similar sexual identity.

A further confirmed finding is that maternal uncles of male homosexuals have approximately twice the incidence of same-sex orientation in comparison to paternal uncles of the same individuals [22].

Homosexuality and kin selection 

The individual with exclusive same-sex orientation must have diminished reproductive success but the family group or tribe of which that individual was a member had enhanced survival fitness [6]. Hamilton's rule describes mathematically how the loss of individual "survival fitness" may benefit the family group or tribe of which an individual is a member [23]. In this circumstance, Hamilton’s rule is fulfilled. Although the concept of kin selection has been challenged, it remains one of the foundations of modern evolutionary biology [24].

Diminished reproductive success implies that usual Mendelian inheritance is unlikely, although a significant linkage between polymorphic markers on the X chromosome and homosexual orientation has been demonstrated [22]. What may be inherited universally in humans is an innate chance possibility, a random occurrence that any individual within a group may have a same-sex orientation.

Concordance in twins

A paradox in our understanding of the biology of homosexuality is the occurrence of same-sex orientation in twins. If homosexuality was entirely genetically determined, MZ twins would always be expected to have identical sexual orientations. This is not the case. MZ twins have been found to have a concordance of 65.8%; if one twin has a non-heterosexual sexual orientation there is an approximately two in three chance that the second of that pair will have that same orientation [8]. DZ twins have been shown to have a concordance for same-sex sexual orientation of 30%, a similar incidence to that of non-twin siblings [9]. The FBO data also indicate that non-genetic factors can influence the development of same-sex orientation.

Inheritance of mitochondria and mitochondrial disorders

The mature ovum contains up to one hundred thousand mitochondria, arranged circumferentially around the cell nucleus [25]. The few mitochondria present in the sperm do not survive in the fertilized ovum; in humans, all mitochondria are maternally inherited. During the development of a mature ovum, there is reduction followed by multiplication of mitochondria so that any variation in normal and pathological mitochondrial proportions may be increased [10]. As a result of this and subsequent variation in the cells of the developing embryo, children from the same mother, although inheriting the same pathological or adaptive variant of mtDNA, may differ markedly in the type and severity of symptoms [11].

Hypothesis

**Figure 2:**
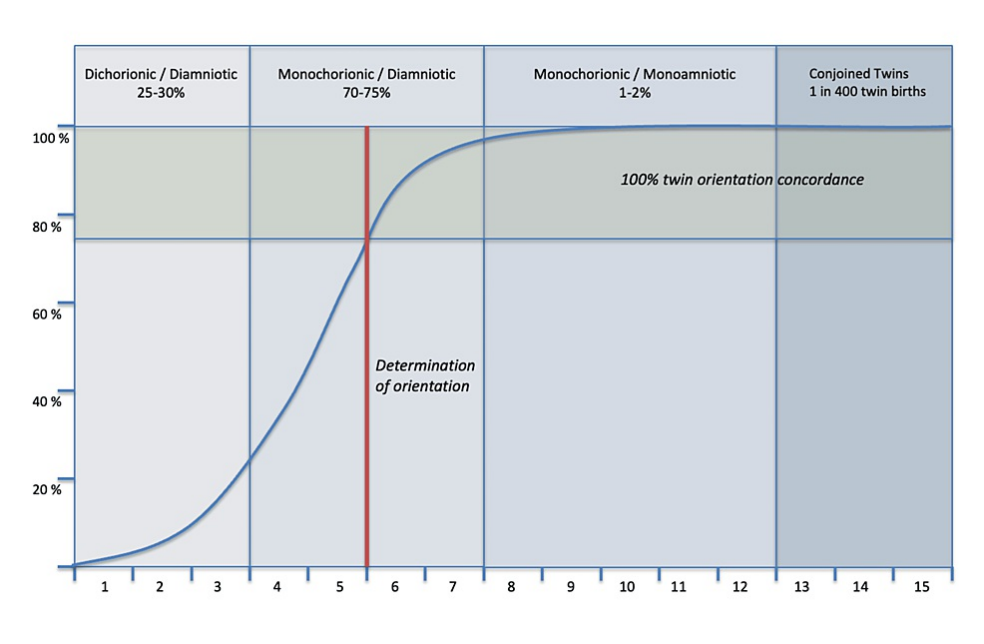
Schematic showing the percentage of identical twin pregnancy splitting occurring over the initial days of embryonic development Identical twins resulting from splitting occurring before day five are free to develop their own sexual orientation. If one twin has a same-sex orientation, there is a 30% chance that the second twin will have this same identity. If splitting occurs after day five, twins have the same sexual orientation. X-axis: days after fertilization, Y-axis: percentage of split embryos (Schematic diagram kindly provided by Dr. Ken Doig, bioinformatician, The University of Melbourne).

The explanation for some differences between the two individuals from an MZ twin pregnancy may relate to the time of embryonic cleavage. Early cleavage allows for more separate developments, later cleavage results in closer identities. It will be proposed that sexual orientation is one characteristic determined early in fetal development, at a stage when the fetus is still in the initial morula or blastocyst stages. MZ twins resulting from separation before day four (DCDA twins) are free to develop their own chorions, as well as other differences including their own sexual identity. However, splitting will have occurred only in 25% to 30% of MZ twins (Figure 2). By day five splitting will have occurred in 70% of embryos. It is proposed that concordance in this early separation group, those splitting on or before day five, should be the same or similar to that of DZ twins or non-twin siblings (30%). If sexual orientation is determined on or about day five, twins splitting after that time should always have the same orientation. It follows that if one twin in this later splitting group should have a non-heterosexual sexual orientation, there is a 100% chance the twin sibling will have the same inclination. Thus 30% of the 70% early splitting group should have orientation concordance; 100% of the later splitting group have identical sexual orientation. The calculation gives an overall concordance rate of 55.5%, close to the observed percentage of 65.8% [8].

A problem with this proposal is that at day five, the embryo is still in the blastocyst stage with no cytological evidence of cellular differentiation; embryonic structures are still to form [25]. Any change that occurs must occur in progenitor cells. At this time there is down-regulation of the maternal genes and up-regulation of embryonic genes [26,27]. A large overrepresentation of genes annotated to developmental processes, multicellular organismal processes, system development, blood vessel development, organ morphogenesis, and brain development was found among genes that were expressed at higher levels in these embryos. These findings suggest that genes regulating further embryonal development are already active at this blastocyst stage. Furthermore, pluripotent embryonic stem cell lines in cell cultures have been derived from human blastocysts. Neural progenitor cells may be isolated from these cell cultures and induced to form mature neurons; thus the presence of these cells has been confirmed [28]. A further event, occurring at all phases of fetal development, is the random flow of mitochondria into daughter cells with the potential for uneven distribution of any mitochondria with adaptive DNA [29].

Discussion

Determination of sexual identity at or about five days after fertilization provides a possible explanation for the level of concordance that has been found in monozygous twins. As embryonic structures are still to form, this determination must occur in progenitor cells, cells that have been shown to be present at this stage of development [28]. Determination of sexual identity, although it may be influenced by other factors, appears to be largely a random event [20,21].

A random event in the field of cellular biology at this stage of embryonic development is the flow of mitochondria into dividing cells [29]. If wild-type mitochondria constitute less than 100%, with adaptive variants present in the mitochondrial genome, the numbers of this variant will differ with differing degrees of heteroplasmy in the daughter cells. Such an occurrence provides a possible explanation for the chance of occurrence of same-sex orientation with gradation of such orientation relating to actual adaptive mtDNA levels. The presence of such adaptive mtDNA is unproven but mtDNA polymorphisms are universal and, as stated, have been shown to influence mitochondrial matrix pH and intracellular functions [13].

The proposal that mitochondrial participation in human sexual orientation at an early stage of embryonic development is supported by the finding that maternal uncles of male homosexuals have approximately twice the incidence of same-sex orientation as do paternal uncles [20,21]. Matrilineal inheritance is also apparent in the children of same-sex couples; a male child conceived by sperm donation to one member of a lesbian partnership is more likely to be homosexual [30]. The reverse does not occur; a child of male sex couples conceived with a sperm donation from one member to a surrogate mother has no increased probability of same-sex orientation. Expressed differently, if a child receives mitochondria from a same-sex orientated female parent that child is more likely to have same-sex orientation; if the child receives mitochondria from a heterosexual mother there is no increase in same-sex orientation even though that child may have had a homosexual father. Mitochondrial-based sex orientation determination is compatible with the maternal immune hypothesis for the FBO effect with any response directed toward mtDNA-derived antigen rather than antigen derived from nDNA [31].

A hypothesis is of no usefulness if it cannot be tested; it has greater validity if it withstands attempted disproval [32]. The proposal that sexual orientation occurs at or before five days after fertilization is capable of testing and disproval. The placental and membrane structure of twin pregnancies is now readily determined by prenatal ultrasonography [33]. If the hypothesis is correct, DCDA MZ twins may independently develop hetero- or same-sex sexual orientation; the frequency of same-sex orientation in this group should be much the same whether twins are MZ or DZ. MCDA twins should have a much higher incidence of concordance but not 100% (due to some splitting occurring on day five). MCMA twins, however, resulting from splitting after day eight, should have 100% concordance. Should MCMA twins develop truly differing sexual orientations, this hypothesis is invalidated and may be safely discarded.

It is not proposed that fetal membrane development is in any way directly connected with the development of human sexuality. Chorionicity of twin pregnancy is simply a measurement of the timing of MZ twin division and it is this timing that appears crucial to subsequent adult sexual orientation. 

Gender dysphoria, although an entirely different condition to same-sex attraction, shows more congruence in MZ twins than DZ twins with similar congruence levels, indicating that this too may be determined at an early phase of fetal development [34]. This same or similar timing may also provide an explanation for the partial concordance of lateralization that occurs in MZ twins [35]. Regardless of any timing, the random flow of mitochondria, mtDNA, and adaptive variant mtDNA into dividing cells remains a possible mechanism for individual differences in twin siblings. How adaptive mitochondria may produce profound changes in cerebral function is not addressed here. However, mtDNA polymorphisms have been shown to influence mitochondrial matrix pH and intracellular biochemical function [13]. These cellular changes in turn may impact cerebral neuronal distribution, axon connections, and communication [36].

A maternal inheritance pattern supports the concept of a mitochondrial-based process in the determination of sexual orientation and explains the increased same-sex orientation in maternal uncles of gay men. This explanation proposes differing levels of inheritance of mitochondria with adaptive mtDNA, occurring as a consequence of varying levels in the original ovum. Further differentiation occurs as the dividing cells in the blastocyst receive differing proportions of this variant at each cell division. Neural progenitor cells may then range widely in their variant heteroplasmy levels; the resultant adult human will later also exhibit a range of sexual inclinations, a level that may be imprecisely assessed on the Kinsey scale. A primary intra-natal explanation for human sexual orientation does not exclude subsequent societal influences. Clearly, an individual with less pivotal inclination is more likely to be affected by circumstances, positive or negative.

As Popper noted, for any theory it is easy to find further confirmation [32]. If there is variation in the incidence of same-sex sexual orientation, such variation may be found in the different mtDNA haplogroups rather than in racial or societal groupings. A "gay gene," if such there be, may well be embedded in our mtDNA rather than our nDNA. There is no evidence supporting the concept that there is adaptive DNA in the mitochondrial genome, which could influence sexual identity, and that is surely the weakest point in this proposal. However, comprehensive mtDNA testing is rapidly advancing and is now capable of demonstrating any such adaptive variants [37]. However, regardless of sexuality preferences or mitochondrial involvement, a general dictum should be the later a developing embryo splits to form an MZ twin pair, the more likely these twins will have shared characteristics.

## Conclusions

Biological determination of human sexual orientation on or about day five after fertilization provides an explanation for the variation of sexual orientation in MZ twins. The examination of twin fetal membranes provides an estimate of the time of splitting of the developing twin embryo and is thus a guide to the probability of concordance of sexual orientation.

Mitochondrial participation, specifically through an unverified adaptive variant of mtDNA, offers a potential explanation for the matrilineal bias observed in human sexual orientation. The variable inheritance and subsequent random distribution of mitochondria containing adaptive mtDNA during blastocyst cell division constitute a plausible biological mechanism that contributes to the broad spectrum and diversity of human sexual orientation. It is important to note that this proposal for the early determination of sexual orientation does not negate the possible influence of later fetal or post-natal factors.

These hypotheses provide avenues for validation, and more significantly, there is a means of disproval. This review should be seen as a novel proposal that incorporates established findings and provides a further explanatory concept for consideration and testing.
